# Blockade Effects of Anti-Interferon- (IFN-) *γ* Autoantibodies on IFN-*γ*-Regulated Antimicrobial Immunity

**DOI:** 10.1155/2019/1629258

**Published:** 2019-05-30

**Authors:** Dyah Ika Krisnawati, Yung-Ching Liu, Yuarn-Jang Lee, Yun-Ting Wang, Chia-Ling Chen, Po-Chun Tseng, Ting-Jing Shen, Chiou-Feng Lin

**Affiliations:** ^1^Graduate Institute of Medical Sciences, College of Medicine, Taipei Medical University, Taipei 110, Taiwan; ^2^Department of Microbiology and Immunology, School of Medicine, College of Medicine, Taipei Medical University, Taipei 110, Taiwan; ^3^Dharma Husada Nursing Academy, Kediri, East Java, Indonesia; ^4^Department of Internal Medicine, School of Medicine, College of Medicine, Taipei Medical University, Taipei 110, Taiwan; ^5^Division of Infectious Diseases, Department of Internal Medicine, Shuang Ho Hospital, Taipei Medical University, Taipei 110, Taiwan; ^6^Division of Infectious Diseases, Department of Internal Medicine, Taipei Medical University Hospital, Taipei 110, Taiwan; ^7^School of Respiratory Therapy, College of Medicine, Taipei Medical University, Taipei 110, Taiwan; ^8^Pulmonary Research Center, Wan Fang Hospital, Taipei Medical University, Taipei 110, Taiwan; ^9^Cell Physiology and Molecular Image Research Center, Wan Fang Hospital, Taipei Medical University, Taipei 110, Taiwan; ^10^Research Center of Thoracic Medicine, Taipei Medical University, Taipei 110, Taiwan

## Abstract

The interferon- (IFN-) *γ* expression is elicited in response to microbial infections and activates immune surveillance by antimicrobial immune elements to induce microbial killing. Patients with adult-onset immunodeficiency who suffer from recurrent infections with microbes, particularly nontuberculous mycobacteria (NTM), commonly display genetic defects in IFN-*γ* signaling as well as the generation of anti-IFN-*γ* autoantibodies (autoAbs). Because IFN-*γ* is an activator of macrophage differentiation and a proinflammatory activator of innate immunity, the blockade effects of the autoAbs present in NTM patient serum on IFN-*γ* are hypothesized to regulate the antimicrobial function of macrophages. In the presence of patient serum, IFN-*γ*-induced type 1 macrophage (M1) differentiation was inhibited in PMA-stimulated human monocytic THP-1 cells. Treatment with patient serum significantly blocked the production of proinflammatory factors, including cytokines/chemokines and reactive oxygen/nitrogen species, by M1 macrophages. Importantly, IFN-*γ*-facilitated phagocytosis and degradation of heat-killed mycobacterium were decreased by cotreatment with patient serum. These results show the blockade activity of anti-IFN-*γ* autoAbs on IFN-*γ*-mediated antimicrobial immunity in macrophages.

## 1. Introduction

The killing of intracellular pathogens by interferon- (IFN-) *γ*-activated macrophages has been linked to the maintenance of antimicrobial immunity in both innate and adaptive immunity [1]. Mycobacteria are intracellular pathogens of macrophages that escape the bactericidal effectors within macrophages directly by interfering with phagosome-lysosome fusion and indirectly by causing immunosuppressive responses [2]. Unfortunately, patients with adult-onset immunodeficiency, which is characterized by defects in IFN-*γ* signaling commonly caused by the generation of anti-IFN-*γ* autoantibodies (autoAbs) and partly due to inherited mutations in IFN-*γ*-signaling-associated factors, usually acquire a variety of bacterial infections, such as infections with *Mycobacterium tuberculosis* (*M. tuberculosis*), nontuberculous mycobacteria (NTM), *Cryptococcus neoformans*, *Penicillium marneffei*, and nontyphoidal *Salmonella* spp. [3, 4]. In these cases, the main pathogenic processes causing disease are still under investigation, but defects in the IFN-*γ*-mediated immune surveillance of mycobacterium infections are generally involved [5].

In immune defense, IFN-*γ* acts as an immunoregulator to elicit antibacterial responses, including phagocytosis, oxidative killing, cell death, and proinflammatory cytokine/chemokine production [6]. Treatment with IFN-*γ* increases the efficiency of the internalization and nonoxidative early intracellular killing of *S. enterica* by human macrophages and modifies the subsequent cytokine release [7]. To enhance intracellular killing abilities, IFN-*γ* activates infected macrophages and induces microbicidal functions via the production of nitric oxide (NO) and reactive oxygen species (ROS). Mechanistically, IFN-*γ* causes activation of the nicotinamide adenine dinucleotide phosphate-dependent phagocyte oxidase system (respiratory burst) through the transcriptional stimulation of gp91^phox^ and p67^phox^ expression, priming NO production, depleting tryptophan, and upregulating lysosomal enzymes that promote microbe destruction [6]. The induction of inducible NO synthase (iNOS)/NO biosynthesis-mediated cell apoptosis by IFN-*γ* acts as a defense mechanism against *M. tuberculosis* in activated macrophages [8].

Improvements in the methodology for detecting anti-IFN-*γ* autoAbs strongly show a link between autoAb production and defects in antimicrobial IFN-*γ* expression in patients with adult-onset immunodeficiency who have active opportunistic infections [9]. Several studies have further shown the association of the human leukocyte antigen- (HLA-) DRB1 and HLA-DQB1 alleles with the generation of anti-IFN-*γ* autoAbs in patients with NTM infections [10–12]. A molecular mimicry-based immunopathogenesis caused by a fungal infection is proposed for the development of anti-IFN-*γ* autoAbs [13]. Although most studies have shown the neutralizing activity of anti-IFN-*γ* autoAbs against the IFN-*γ*-activated signal transducer and activator of transcription 1 (STAT1), the pathological effects of anti-IFN-*γ* autoAbs showing a counteracting role in IFN-*γ*-mediated antimicrobial activity are not fully addressed. To show that the speculation is correct, in this project, we assessed the blocking effect of anti-IFN-*γ* autoAbs on monocyte-derived type 1 macrophage (M1) differentiation, the production of NO and ROS, and the mycobacterial killing capability of macrophages.

## 2. Materials and Methods

### 2.1. Serum

Blood samples were obtained from three healthy controls and three patients according to the guidelines established by the institutional review board of the Taipei Medical University Hospital (N201804015). Patients with a clinical diagnosis of adult-onset immunodeficiency, including disseminated infections with opportunistic pathogens, NTM infections, and HIV-negative status, were enrolled in the study, and the diagnosis of anti-IFN-*γ* autoAbs has been defined previously [14]. Briefly, a modified sandwich ELISA, adapted from a commercial human IFN-*γ* sandwich ELISA kit (R&D Systems, Minneapolis, MN), was used to detect anti-IFN-*γ* autoAbs in the study. In capturing anti-human IFN-*γ*-coated microwells, the wells were blocked with 0.5% bovine serum albumin (BSA) in phosphate-buffered saline (PBS) Tween 20 buffer and then recombinant human IFN-*γ* and tested sera were added. The binding of anti-IFN-*γ* autoAbs was measured by HRP-conjugated detection Abs against human IgG according to the manufacturer's instructions.

### 2.2. Reagents and Abs

Human IFN-*γ* was purchased from PeproTech (Rocky Hill, NJ). All of the reagents used in this study were obtained from Sigma-Aldrich (St Louis, MO) and Tocris Bioscience (Bristol, UK). Alexa Fluor 488- and horseradish peroxidase- (HRP-) conjugated goat anti-rabbit and anti-human IgG antibodies were obtained from Chemicon International (Temecula, CA) and Jackson ImmunoResearch laboratories Inc. (West Grove, PA).

### 2.3. Cell Culture

Human monocytic THP-1 cells (ATCC, TIB-202) were grown and maintained in a 10 cm plate in RPMI 1640 medium (RPMI; Invitrogen Life Technologies, Rockville, MD), with L-glutamine and supplemented with 10% heat-inactivated fetal bovine serum (Invitrogen Life Technologies), 50 units of penicillin, and 50 mg/ml of streptomycin.

### 2.4. Monocyte-Derived Macrophages

THP-1 monocytes were differentiated into macrophages (M0) by 72 h incubation with 150 nM phorbol 12-myristate 13-acetate (PMA, Sigma-Aldrich) followed by 24 h incubation in RPMI medium. The macrophages were polarized into M1 macrophages by incubating with 20 ng/ml IFN-*γ* (R&D Systems, Minneapolis, MN) for 24 h.

### 2.5. Immunostaining

Cells were fixed with 4% paraformaldehyde, permeabilized with 0.5% Triton X-100, and washed twice with ice-cold PBS. Cells were stained with Abs against CD68, CD14, and CD86 and then stained with an Alexa Fluor 488-conjugated goat anti-human IgG antibody. 4′,6-Diamidino-2-phenylindole (DAPI, 5 *μ*g/ml) was used for nuclear staining. Cells were visualized under a fluorescence microscope (BX51; Olympus, Tokyo, Japan) and/or a FACSCalibur flow cytometer (BD Biosciences, San Jose, CA).

### 2.6. Cytokine/Chemokine Detection

The concentrations of TNF-*α* and IP-10 in the cell-conditioned culture medium and serum were determined using ELISA kits (RD Systems) according to the manufacturer's instructions.

### 2.7. Western Blot Analysis

Total lysates were separated using SDS-polyacrylamide gel electrophoresis and then transferred to a polyvinylidene difluoride membrane (Millipore Corporation, Billerica, MA). After blocking with 5% BSA, the cut blots were developed with a one to one thousand dilution of the tested serum and the indicated primary Abs. Finally, the blots were hybridized with an HRP-conjugated goat anti-rabbit IgG antibody and developed using an ECL Western blot detection kit (Millipore Corporation).

### 2.8. Griess Reaction

The production of nitric oxide (NO) was assessed as the accumulation of nitrite (NO^2-^) in the medium using a colorimetric reaction with a Griess reagent.

### 2.9. ROS Detection

Cells were exposed to 10 *μ*M CM-H_2_DCFDA (Invitrogen, San Diego, CA) for 5 min. The cells were then analyzed using the FL-1 channel (515-545 nm) of a FACSCalibur flow cytometer (BD Biosciences). The mean fluorescence intensity (MFI) was analyzed using CellQuest Pro 4.0.2 software, and quantification was performed using WinMDI 2.8 software (The Scripps Research Institute, La Jolla, CA). Small cellular debris was excluded by gating on a forward-scatter plot.

### 2.10. Phagocytosis

One vial of heat-killed *Mycobacterium tuberculosis* (HKMT, InvivoGen, San Diego, CA) was diluted with 1 ml double-distilled H_2_O and stained with 1/100 SYTO 16 Green (Invitrogen, Eugene, OR) for 0.5 h. Phagocytosis was detected by using a FACSCalibur flow cytometer (BD Biosciences).

### 2.11. Statistical Analysis

Values are expressed as the mean ± standard deviation (SD). Groups were compared by using Student's two-tailed unpaired *t*-test or one-way ANOVA followed by Dunnett's post hoc test, as appropriate. These analyses were performed by using GraphPad Prism 4 software (GraphPad Software, La Jolla, CA). Statistical significance was set at *p* < 0.05.

## 3. Results

### 3.1. IFN-*γ* AutoAb-Positive Patient Serum Blocks IFN-*γ*-Driven M1 Macrophage Differentiation

**Figure 1:**
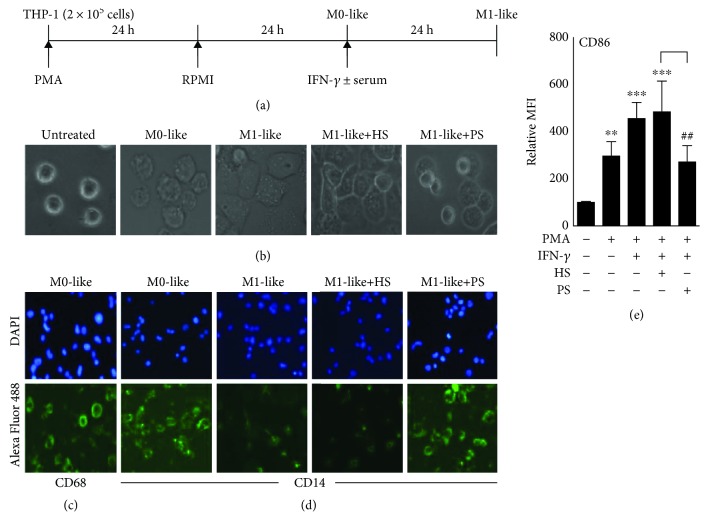
Blocking effects of anti-IFN-*γ* autoAbs on monocyte-derived M1 macrophages. Human monocytic THP-1 cells were treated with PMA to induce cell differentiation toward a macrophage-like (M0) morphological phenotype (a) with increased adherent morphology (b) and expression of CD68 (c). Following IFN-*γ* treatment in the presence of anti-IFN-*γ* autoAb-positive patient serum (PS) or anti-IFN-*γ* autoAb-negative healthy serum (HS), immunostaining followed by fluorescence microscopy or flow cytometry showed the expression of M1-like macrophage biomarkers, such as CD14 (d) and CD86 (e). All immunostaining data are shown as one representative image. DAPI was used for nuclear staining. Relative mean fluorescence intensity (MFI) was normalized in the untreated group. ^∗∗^*p* < 0.01 and ^∗∗∗^*p* < 0.001 compared with the control group. ^##^*p* < 0.01 compared with the HS group.

An ELISA-based detection assay for anti-IFN-*γ* autoAbs has been developed [3], and we previously identified patients with adult-onset immunodeficiency by characterizing the generation of anti-IFN-*γ* autoAbs [14]. To confirm the functional neutralizing activity of these autoAbs, particularly against antimicrobial IFN-*γ*, the blockade of IFN-*γ*-driven M1 macrophage differentiation and polarization, which are classically activated in response to microbial pathogens [15, 16], was evaluated. In this study, phorbol 12-myristate 13-acetate- (PMA-) driven human monocytic THP-1 cell differentiation into macrophages was performed and confirmed by the changes in cell morphology (Figures 1(a) and 1(b)) and increased CD68 expression [17] (Figure 1(c)). Following IFN-*γ* stimulation, M1 macrophage differentiation occurred, as indicated by decreased CD14 (Figure 1(d)) and increased CD86 (Figure 1(e)) expressions [18], while treatment with the patient serum (PS), which contains anti-IFN-*γ* autoAbs, effectively blocked these IFN-*γ*-mediated effects. Healthy serum (HS) is used as the control group. These data demonstrate the inhibitory effects of anti-IFN-*γ* autoAbs on IFN-*γ*-driven M1 macrophage differentiation.

### 3.2. IFN-*γ* AutoAb-Positive Patient Serum Attenuates IFN-*γ*-Induced Cytokine and Chemokine Production as well as iNOS/NO Biosynthesis and ROS Generation in Differentiated Macrophages

**Figure 2:**
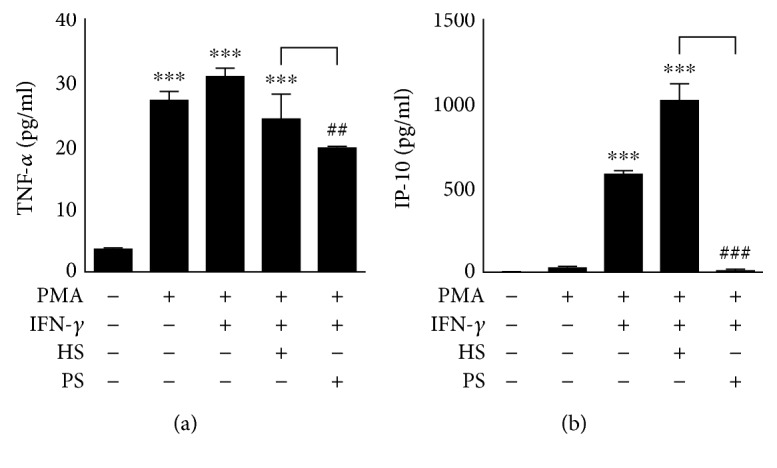
Treatment with anti-IFN-*γ* autoAbs decreases IFN-*γ*-induced cytokine and chemokine production in differentiated macrophages. After treatment with anti-IFN-*γ* autoAb-positive patient serum (PS) or anti-IFN-*γ* autoAb-negative healthy serum (HS), ELISAs showed the production of (a) TNF-*α* and (b) IP-10 by IFN-*γ*-stimulated PMA-differentiated macrophages. The quantitative data are shown as the mean ± SD from three independent experiments. ^∗∗∗^*p* < 0.001 compared with the control group. ^##^*p* < 0.01 and ^###^*p* < 0.001 compared with the HS group.

**Figure 3:**
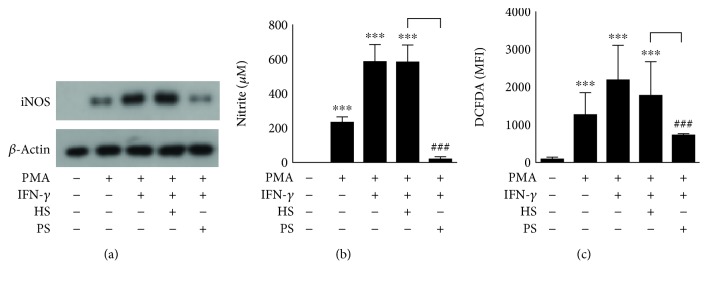
Stimulation with anti-IFN-*γ* autoAbs decreases IFN-*γ*-induced generation of nitrite and hydrogen peroxide in differentiated macrophages. After treatment with anti-IFN-*γ* autoAb-positive patient serum (PS) or anti-IFN-*γ* autoAb-negative healthy serum (HS), (a) Western blot analysis, (b) Griess reaction, and (c) CM-H_2_DCFDA staining showed the biosynthesis of iNOS, the biosynthesis of NO, and the generation of hydrogen peroxide in IFN-*γ*-stimulated PMA-differentiated macrophages, respectively. The quantitative data are shown as the mean ± SD from three independent experiments. For DCFDA detection, the stained cells were analyzed using a flow cytometer and the data are shown as the mean fluorescence intensity (MFI). ^∗∗∗^*p* < 0.001 compared with the control group. ^###^*p* < 0.001 compared with the HS group.

IFN-*γ* induces cytokine and chemokine production as well as iNOS/NO biosynthesis and ROS generation in macrophages, which are all processes related to IFN-*γ* antimicrobial bioactivities [6–8, 19]. By using an inflammatory cell model of IFN-*γ*-induced activation in human monocytic THP-1 cell-derived macrophages, the production of the cytokine tumor necrosis factor- (TNF-) *α* and the chemokine C-X-C motif chemokine 10, also known as IFN-*γ*-induced protein- (IP-) 10, as well as iNOS/NO biosynthesis and ROS generation, was monitored in the presence or absence of anti-IFN-*γ* autoAb-positive patient serum. Compared with the healthy control serum, the patient serum significantly (*p* < 0.01) blocked the IFN-*γ*-induced production of TNF-*α* (Figure 2(a)) and IP-10 (Figure 2(b)) as well as the expression of iNOS (Figure 3(a)) and generation of NO (Figure 3(b)) and ROS (Figure 3(c)). These results demonstrate the neutralizing activity of anti-IFN-*γ* autoAbs against the IFN-*γ*-induced upregulation of antimicrobial factors in differentiated macrophages.

### 3.3. Blockade Effects of IFN-*γ* AutoAbs on IFN-*γ*-Induced Phagocytosis in Differentiated Macrophages

**Figure 4:**
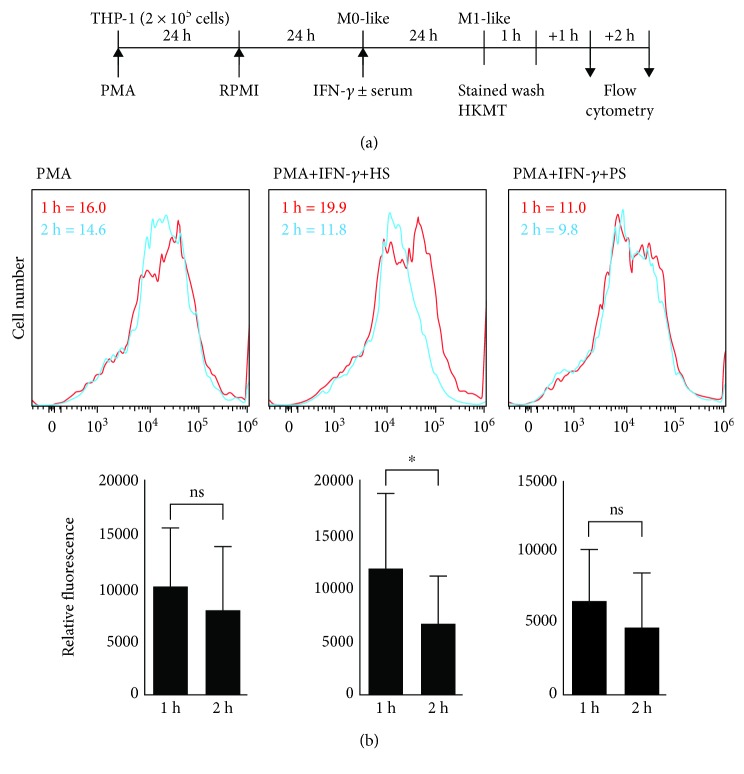
Anti-IFN-*γ* autoAbs reduce the IFN-*γ*-induced bacterial killing capability of differentiated macrophages. (a) PMA-differentiated macrophages were treated with IFN-*γ* in the presence or absence of anti-IFN-*γ* autoAb-positive patient serum (PS) and incubated with SYTO 16 Green-stained heat-killed *M. tuberculosis* (HKMT). (b) Following the flow cytometry analysis, bacterial killing capability was analyzed by measuring decreases in the mean fluorescence intensity (MFI). All histograms show representative data. ns: not significant. ^∗^*p* < 0.05.

IFN-*γ* facilitates innate immunity by triggering the internalization of bacteria through a mechanism involving phagocytosis and intracellular killing within phagolysosomes [6, 7]. Following phagocytosis, IFN-*γ* promotes intracellular acidification, autophagy, and oxidative stress for bactericidal execution [1, 5, 20]. To validate the blockade effect of anti-IFN-*γ* autoAbs on IFN-*γ*-facilitated phagocytosis, fluorescently labeled heat-killed *M. tuberculosis* (HKMT) bacteria were utilized according to a previously published protocol [21] to evaluate the phagocytic activity of IFN-*γ*-activated M1 macrophages (Figure 4(a)). Compared with the healthy control serum, the patient serum significantly (*p* < 0.05) blocked IFN-*γ*-induced eradication of fluorescently labeled HKMT (Figure 4(b)). These results demonstrate the blockade effect of anti-IFN-*γ* autoAbs on IFN-*γ*-induced mycobacterial phagocytosis in differentiated macrophages.

## 4. Discussion

Patients with adult-onset immunodeficiency have been identified by characterizing defects in IFN-*γ* signaling, which generally involve the generation of anti-IFN-*γ* autoAbs and are partly due to inherited mutations in IFN-*γ*-signaling-associated factors [3, 4]. While the detection methodology has been demonstrated and modified [3, 22, 23], a functional neutralizing assay is lacking, although an assessment of IFN-*γ*-activated STAT1 is usually carried out. Our study found blockade effects of anti-IFN-*γ* autoAbs not only on the IFN-*γ* signaling pathway [14] but also on IFN-*γ*-driven antimicrobial activity, including M1 macrophage differentiation, antimicrobial factor production, and phagocytic activation. The generation of anti-IFN-*γ* autoAbs, as with the IFN-*γ* genetic defects, increases the immunosuppression of patients facing infectious pathogens [3, 4]. In this situation, the progression of adult-onset immunodeficiency increases the possibility that these patients will develop recurrent opportunistic pathogen infections due to the nonfunctional antimicrobial IFN-*γ*.

Phagocytes are essential for eliminating infectious microorganisms and for presenting the possible epitopes to cells of the adaptive immune system [2, 15, 16]. Professional M1 macrophages are functionally polarized and classically activated in response to microorganisms and host mediators, particularly IFN-*γ* [15]. In general, activated M1 macrophages are able to produce proinflammatory cytokines/chemokines, phagocytize microbes, generate bactericidal NO and ROS, and initiate an immune response [16]. In this study, we first demonstrated the blockade effects of anti-IFN-*γ* autoAbs on the IFN-*γ*-driven polarization and activation of M1 macrophages. The results show that retarding M1 macrophage development with anti-IFN-*γ* autoAbs makes possible the inhibition of IFN-*γ*-driven antimicrobial processes, including efficient internalization; expression of antimicrobial factors, including cytokines/chemokines, iNOS/NO, and ROS; and efficient phagocytic eradication. These results strongly support the pathological blockade effects of anti-IFN-*γ* autoAbs on antimicrobial activities driven by IFN-*γ*.

Regarding the limitations on early diagnosis, potential treatment is relatively emerging for controlling disease onset and/or progression of adult-onset immunodeficiency [3, 4]. Treatment of antibiotic linezolid for disseminated nontuberculous mycobacterial infection in anti-IFN-*γ* autoAb-positive patients shows 50% complete resolution of signs and symptoms [24]. Interestingly, treatment of rituximab, a chimeric monoclonal antibody against the protein CD20 on B cells, has ameliorated disseminated *M. avium* infection in a patient with anti-IFN-*γ* autoAb by recovering IFN-*γ* signaling [25]. Currently, the use of immunotherapy with pulse intravenous cyclophosphamide therapy has been reported to be an alternative treatment against adult-onset immunodeficiency patients infected with *M. abscessus* [26]. Either targeting mycobacterial infection or restoring IFN-*γ* antimicrobial bioactivities is the key point for treating mycobacterial infection in patients with adult-onset immunodeficiency.

Although the generation of anti-IFN-*γ* autoAbs is highly associated with the development of disseminated NTM infection in patients without HIV-induced immunodeficiency [4], the levels and pathogenic roles of anti-IFN-*γ* autoAbs in the progression of adult-onset immunodeficiency are still unclear. Based on the current tests for measuring anti-IFN-*γ* autoAb expression, possible epitopes related to IFN-*γ* bioactivity have been selected for validation [13, 27]. The possible targeting effects retarding IFN-*γ* bioactivity need further investigation. Furthermore, verifying the levels of neutralizing anti-IFN-*γ* autoAbs associated with the severity in adult-onset immunodeficiency is necessary for characterizing its pathogenic role. Although our present data showed the possible inhibition of anti-IFN-*γ* autoAbs on IFN-*γ*-mediated antimicrobial activities, their pathogenic role needs further validation by using the suitable *in vitro* and *in vivo* models of bacterial infection. In conclusion, this study validated the blockade effects of IFN-*γ* autoAbs on the IFN-*γ*-driven polarization and activation of M1 macrophages. Furthermore, IFN-*γ*-induced antimicrobial factors and phagocytic processes were also decreased by the presence of patient serum containing anti-IFN-*γ* autoAbs.

## 5. Conclusions

The results not only provide possible standardized tests for assessing the functional neutralizing activity of the anti-IFN-*γ* autoAbs present in patient serum but also have implications on the pathogenesis of adult-onset immunodeficiency resulting from the blockade of antimicrobial IFN-*γ*. In support of the established methods, samples are needed for further validation with their clinical outcome related to the levels of pathogenic neutralizing anti-IFN-*γ* autoAbs.

## Data Availability

The data used to support the findings of this study are available from the corresponding author upon request.

## References

[1] Murray H. W. (1994). Interferon-gamma and host antimicrobial defense: current and future clinical applications. *The American Journal of Medicine*.

[2] Pieters J. (2008). *Mycobacterium tuberculosis* and the macrophage: maintaining a balance. *Cell Host & Microbe*.

[3] Browne S. K., Burbelo P. D., Chetchotisakd P. (2012). Adult-onset immunodeficiency in Thailand and Taiwan. *The New England Journal of Medicine*.

[4] Browne S. K. (2014). Anticytokine autoantibody–associated immunodeficiency. *Annual Review of Immunology*.

[5] Flynn J. L., Chan J., Triebold K. J., Dalton D. K., Stewart T. A., Bloom B. R. (1993). An essential role for interferon gamma in resistance to Mycobacterium tuberculosis infection. *Journal of Experimental Medicine*.

[6] Schroder K., Hertzog P. J., Ravasi T., Hume D. A. (2004). Interferon-*γ*: an overview of signals, mechanisms and functions. *Journal of Leukocyte Biology*.

[7] Gordon M. A., Jack D. L., Dockrell D. H., Lee M. E., Read R. C. (2005). Gamma interferon enhances internalization and early nonoxidative killing of *Salmonella enterica* serovar Typhimurium by human macrophages and modifies cytokine responses. *Infection and Immunity*.

[8] Herbst S., Schaible U. E., Schneider B. E. (2011). Interferon gamma activated macrophages kill mycobacteria by nitric oxide induced apoptosis. *PLoS One*.

[9] Wongkulab P., Wipasa J., Chaiwarith R., Supparatpinyo K. (2013). Autoantibody to interferon-gamma associated with adult-onset immunodeficiency in non-HIV individuals in Northern Thailand. *PLoS One*.

[10] Phoompoung P., Ankasekwinai N., Pithukpakorn M. (2017). Factors associated with acquired anti IFN-*γ* autoantibody in patients with nontuberculous mycobacterial infection. *PLoS One*.

[11] Chi C. Y., Chu C. C., Liu J. P. (2013). Anti-IFN-*γ* autoantibodies in adults with disseminated nontuberculous mycobacterial infections are associated with HLA-DRB1∗16:02 and HLA-DQB1∗05:02 and the reactivation of latent varicella-zoster virus infection. *Blood*.

[12] Pithukpakorn M., Roothumnong E., Angkasekwinai N. (2015). HLA-DRB1 and HLA-DQB1 are associated with adult-onset immunodeficiency with acquired anti-interferon-gamma autoantibodies. *PLoS One*.

[13] Lin C. H., Chi C. Y., Shih H. P. (2016). Identification of a major epitope by anti-interferon-*γ* autoantibodies in patients with mycobacterial disease. *Nature Medicine*.

[14] Krisnawati D. I., Liu Y. C., Lee Y. J. (2019). Functional neutralization of anti-IFN-*γ* autoantibody in patients with nontuberculous mycobacteria infection. *Scientific Reports*.

[15] Benoit M., Desnues B., Mege J. L. (2008). Macrophage polarization in bacterial infections. *The Journal of Immunology*.

[16] Labonte A. C., Tosello-Trampont A.-C., Hahn Y. S. (2014). The role of macrophage polarization in infectious and inflammatory diseases. *Molecules and Cells*.

[17] Daigneault M., Preston J. A., Marriott H. M., Whyte M. K. B., Dockrell D. H. (2010). The identification of markers of macrophage differentiation in PMA-stimulated THP-1 cells and monocyte-derived macrophages. *PLoS One*.

[18] Genin M., Clement F., Fattaccioli A., Raes M., Michiels C. (2015). M1 and M2 macrophages derived from THP-1 cells differentially modulate the response of cancer cells to etoposide. *BMC Cancer*.

[19] Vogel D. Y. S., Glim J. E., Stavenuiter A. W. D. (2014). Human macrophage polarization in vitro: maturation and activation methods compared. *Immunobiology*.

[20] Rovetta A. I., Pena D., Hernandez Del Pino R. E. (2014). IFNG-mediated immune responses enhance autophagy against *Mycobacterium tuberculosis* antigens in patients with active tuberculosis. *Autophagy*.

[21] Doig C., Seagar A. L., Watt B., Forbes K. J. (2002). The efficacy of the heat killing of *Mycobacterium tuberculosis*. *Journal of Clinical Pathology*.

[22] Rattanathammethee K., Chawansuntati K., Chaiwarith R., Praparattanapan J., Supparatpinyo K., Wipasa J. (2018). Dot enzyme-linked immunosorbent assay strip as a screening tool for detection of autoantibody to interferon gamma in sera of suspected cases of adult-onset immunodeficiency. *Journal of Clinical Laboratory Analysis*.

[23] Shima K., Sakagami T., Tanabe Y. (2014). Novel assay to detect increased level of neutralizing anti-interferon gamma autoantibodies in non-tuberculous mycobacterial patients. *Journal of Infection and Chemotherapy*.

[24] Chetchotisakd P., Anunnatsiri S. (2014). Linezolid in the treatment of disseminated nontuberculous mycobacterial infection in anti-interferon-gamma autoantibody-positive patients. *Southeast Asian Journal of Tropical Medicine and Public Health*.

[25] Koizumi Y., Sakagami T., Nishiyama N. (2017). Rituximab restores IFN-*γ*-STAT1 function and ameliorates disseminated *Mycobacterium avium* infection in a patient with anti-interferon-*γ* autoantibody. *Journal of Clinical Immunology*.

[26] Chetchotisakd P., Anunnatsiri S., Nanagara R., Nithichanon A., Lertmemongkolchai G. (2018). Intravenous cyclophosphamide therapy for anti-IFN-gamma autoantibody-associated *Mycobacterium abscessus* infection. *Journal of Immunology Research*.

[27] Wipasa J., Chaiwarith R., Chawansuntati K., Praparattanapan J., Rattanathammethee K., Supparatpinyo K. (2018). Characterization of anti-interferon-*γ* antibodies in HIV-negative immunodeficient patients infected with unusual intracellular microorganisms. *Experimental Biology and Medicine*.

